# Influence of daily usage times on patients’ compliance during at-home bleaching: a randomized clinical trial

**DOI:** 10.1590/1678-7757-2023-0181

**Published:** 2023-10-09

**Authors:** Caio César PAVANI, Ticiane Cestari FAGUNDES, Daniel SUNDFELD, Gabriela Cristina SANTIN, Lucas Silveira MACHADO, André Pinheiro de Magalhães BERTOZ, Timm Cornelius SCHOTT, Renato Herman SUNDFELD

**Affiliations:** 1 Universidade Estadual Paulista Faculdade de Odontologia de Araçatuba Departamento de Odontologia Preventiva e Restauradora Araçatuba São Paulo Brasil Universidade Estadual Paulista (UNESP), Faculdade de Odontologia de Araçatuba, Departamento de Odontologia Preventiva e Restauradora, Araçatuba, São Paulo, Brasil.; 2 Centro Universitário Uningá Departamento de Odontologia Restauradora e Prótese Dentária Maringá PR Brasil Centro Universitário Uningá – UNINGÁ, Departamento de Odontologia Restauradora e Prótese Dentária, Maringá, PR, Brasil.; 3 Universidade Estadual de Maringá Departamento de Odontologia Maringá PR Brasil Universidade Estadual de Maringá - UEM, Departamento de Odontologia, Maringá, PR, Brasil.; 4 Universidade Federal do Rio Grande do Sul Faculdade de Odontologia Departamento de Odontologia Conservadora Porto Alegre RS Brasil Universidade Federal do Rio Grande do Sul, Faculdade de Odontologia, Departamento de Odontologia Conservadora, Porto Alegre, RS, Brasil.; 5 Winterthur Switzerland Private Orthodontic Practice, Winterthur, Switzerland.

**Keywords:** Carbamide peroxide, Clinical trial, Patient compliance, Tooth bleaching, Bleaching agents, Treatment adherence and compliance

## Abstract

The effectiveness of at-home dental bleaching treatments depends on the time that bleaching products are in contact with the teeth surface and, consequently, on the adequate use of associated custom acetate trays.

**Objective:**

This randomized single-blinded trial aimed to analyze if the daily usage time of these products influences the patient’s compliance behavior when submitted to monitored at-home dental bleaching. Secondary outcomes were color change and tooth sensitivity.

**Methodology:**

Sixty-six volunteers were randomly distributed into three groups (n=22): patients were instructed to use the trays for 2 (G2), 4 (G4), and 8 (G8) hours daily. The daily dental bleaching compliance behavior was measured using a microsensor inserted into the trays. Subjective and objective color evaluation assessments were adopted at baseline (T0), one (T1), two (T2), and three weeks (T3) after the beginning of the bleaching treatment, as well as two weeks after the treatment (T4). Tooth sensitivity was analyzed using the VAS scale, ranging from T1 to T4.

**Results:**

G2 showed a greater degree of cooperation than G8 and cooperation was inversely proportional to the recommended usage time. Significantly higher color change was observed in the upper arch for G8 when compared to G2 in subjective analysis, from T1 to T4. There were no statistical differences between the groups in objective analysis.

**Conclusion:**

Shorter recommended usage time of the bleaching product may improve the patient's compliance with at-home dental bleaching treatments. However, increased daily usage time may promote better subjective color change. Bleaching sensitivity was more significant in the first week for a longer time of use.

## Introduction

The effectiveness of at-home dental bleaching treatments depends on the time that bleaching products are in contact with the teeth surface and, consequently, on the adequate use of custom acetate trays. The home bleaching treatment was introduced in 1989, recommending the use of a 10% carbamide peroxide for 6 to 8 daily hours.^1^ Nowadays, different usage times for home bleaching treatments have been reported in the literature.^2-5^ More than 50% of the active agent in carbamide peroxide is available in custom acetate trays after two hours of use.^6^ For this reason, shorter usage times have been proposed.^3^ However, it is important to mention that at-home dental bleaching procedures using carbamide peroxide products are commonly carried out by patients without supervision, which may lead to incorrect use.^7-9^ Thus, professionals may encounter undesirable clinical results when the patient’s compliance is not optimal, which may lead to the patient being dissatisfied.

Usage time measurements for removable orthodontic appliances have been performed using an electronic device that is able to identify the temperature of the oral cavity, which can be converted into time of use in the oral cavity.^10-12^Case reports produced by our group have described the same method to calculate the daily usage time of the bleaching product by inserting a microsensor in the custom acetate tray.^7-9^The usage time recorded by these devices coupled in trays could certainly be an important tool for professionals to promptly identify and overcome possible cooperation problems, providing more effective clinical results.

In the literature, there is a lack of studies that evaluate the influence of monitored daily usage times in the compliance of patients during at-home bleaching using randomized clinical trials and in color change and sensitivity. The objective of the present study was to assess the patient’s compliance and the tooth color change and sensitivity during at-home dental bleaching procedures with different usage times. The null hypothesis tested was: [1] daily usage time would not influence patients’ compliance during at-home bleaching; [2] daily usage time would not influence tooth color change during at-home bleaching, and [3] daily usage time would not influence tooth sensitivity during at-home bleaching.

## Methodology

### Trial design

This study was a prospective, randomized, parallel, single-blind clinical trial, following the guidelines published by the Consolidated Standards of Reporting Trials-CONSORT^13^ and approved by the Local Ethics Research Committee (#CAAE no 66695717.4.0000.5420). It was also registered in the REBEC database (#RBR-77SBFP). It was conducted at Araçatuba School of Dentistry, a medium-sized city of São Paulo State, from March 2018 to April 2019.

The studied factor was the usage time of the trays at three levels: 2 (G2), 4 (G4), and 8 (G8) hours/day. The response variables were the patient’s compliance behavior regarding the usage time of trays, the efficacy of dental bleaching, assessed with visual and spectrophotometric evaluations, and tooth sensitivity.

#### Sample size

The sample size was calculated using a dedicated statistical software package (IBM SPSS Statistics 22.0; IBM Analytics, Armonk, NY, USA). The primary outcome (patient’s compliance behavior) was not used for sample size calculation due to the lack of data about this topic. One of the secondary outcomes was color change: considering a 2.4 standard deviation from a pilot study and the minimum difference to be detected from 3.3, with a 95% confidence level and a maximum acceptable error of 5%, each group required 20 participants. Considering a possible loss of patients, 10% of the total sample was added. The final calculation was 66 participants, once it is a parallel design trial.

## Eligibility criteria

Patients were selected according to the following inclusion criteria: absence of carious lesions; good oral soft tissue health; absence of unsatisfactory and/or fractured restorations; absence of periodontal diseases; absence of non-carious cervical lesions and exposed dentinal tissue in the incisal areas; no history of adverse reaction to peroxides; shade of canines and incisors teeth should be at least A2 (according to Vita Classical Shade Guide, Bad Sackingen, Germany); absence of teeth with spontaneous pain; aged between 18-30 years.

**Figure 1:**
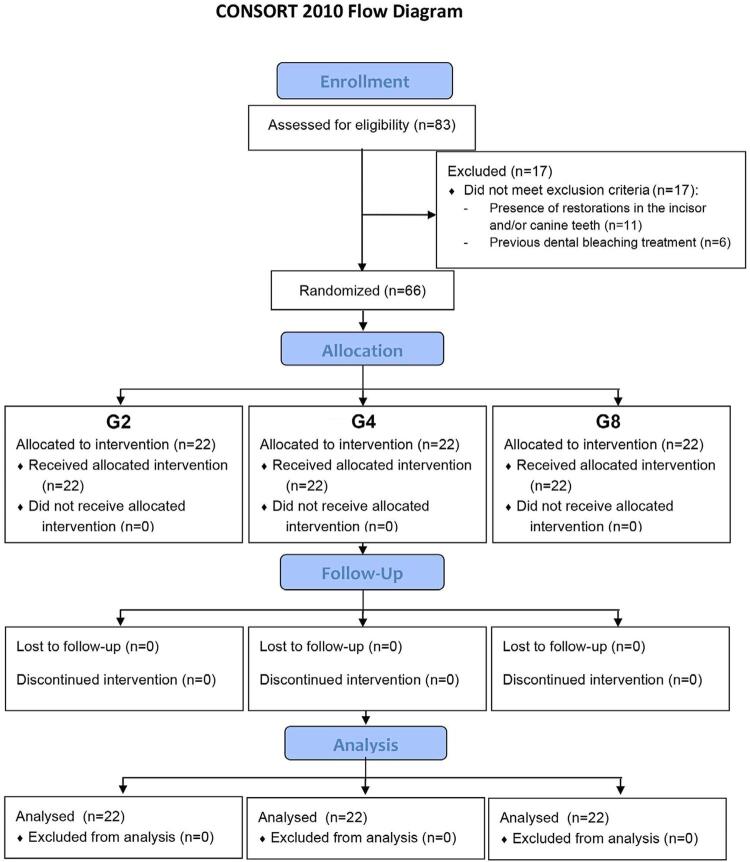
Consort flow-chart of the clinical trial

The exclusion criteria were: presence of proximal and/or vestibular restorations in the incisor and/or canine teeth; teeth with fixed orthodontic appliances and with resinous residues after bracket debonding; pregnancy; breastfeeding; smoking; routine alcohol use; previous dental bleaching treatments; teeth with fluorosis, enamel stains; endodontically treated teeth; bruxism habits; experiencing tooth sensitivity before this study; unacceptance of the research consent form and/or unavailability/lack of commitment to the research (Figure 1).

## Allocation

After selecting the 66 participants, informed consent was obtained from each patient and they were divided into three groups with 22 participants each. Participants’ allocation into these groups was done at an equal rate. The purpose of this allocation was to assign each participant one out of three different usage times, as shown in Figure 1. To ensure impartiality and avoid any bias, each group was labeled with a code (G2, G4, and G8) and codes were placed inside a black opaque envelope. The process of determining which group each volunteer would belong to was conducted through a random draw. This draw was performed by an individual who had no involvement in the study, to maintain objectivity and eliminate any potential influence.

## Bleaching procedure

**Figure 2:**
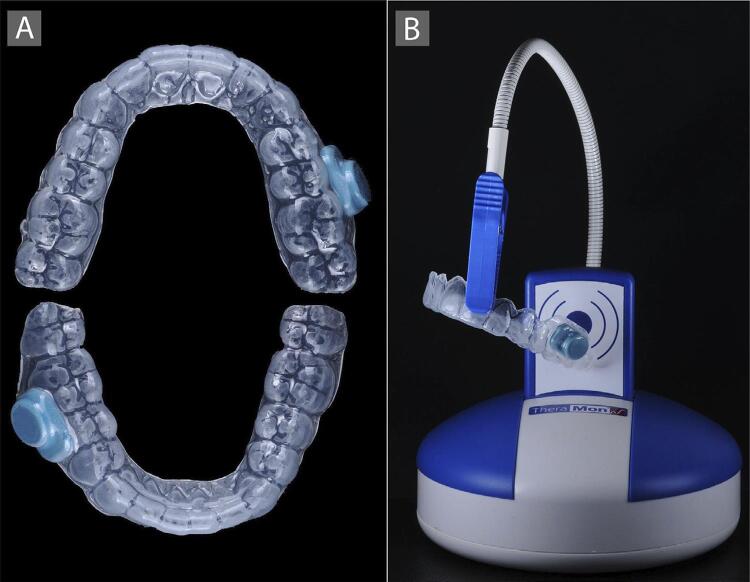
A- Upper and lower acetate trays with the microsensor attached to the buccal surface; B- Reading station for transferring data to the software

Prophylaxis with pumice stone and water was performed. Alginate impressions (Jeltrate Plus, Dentsply, Petrópolis, RJ, Brazil) of each patient’s upper and lower arches were taken and stone models was fabricated. Ethylene vinyl acetate trays (Bio-Art Equip Odontológicos, Ltda, São Carlos, SP, Brazil) were fabricated using a vacuum-pressing machine (Equip Odontológicos Ltda, São Carlos, SP, Brazil). An electronic microsensor (TheraMon microelectronic system, Sales AgencyGschladt, Hargelsberg, Austria) was attached to the buccal surfaces of the acetate trays in order to monitor the daily usage time. The microsensor was included between the 2 acetate plates, each being 1 mm thick, during the preparation of the trays, in which borders were cut 2 mm beyond the gingival margin, following the gingival contour (scalloped tray), without the presence of reservoirs (Figure 2A).

This microsensor is able to measure the temperature of the oral cavity every 15 minutes with a +/- 0.1º Celsius accuracy.^14^ The sensor must be initially activated at the time of the first acquisition, using the associated software (Theramon Software, MC Technology GmbH, Hargelsberg, Austria). The collected data was transferred through a wireless connection between the sensors and a reading station (Figure 2B) coupled to a Universal Serial Bus (USB) connection. The patients were notified about the presence of the microsensors on the acetate trays.

Dental bleaching was performed using 10% carbamide peroxide gel (Opalescense PF, Ultradent Products Inc., South Jordan, UT, USA). Patients were instructed to insert a small drop of bleaching gel on the buccal impression of each tooth on both trays (upper and lower), from the right second premolar to the left second premolar. Patients were instructed to keep the trays on for 2, 4, or 8 hours/day (corresponding to groups G2, G4, and G8, respectively). They were instructed on how and for how long to use the trays by a researcher that was not involved in the implementation and evaluation processes. The dental bleaching technique was performed for 21 consecutive days. Each volunteer’s usage time was obtained and analyzed at the end of the bleaching treatment.

## Daily compliance behavior degree

A daily score for the usage time was created: 0 – patient did not used the trays; 1 – patient used the trays for the recommended time; 2 – patient used the trays for less than the recommended time; 3 – patient used the trays for more than the recommended time. The scores were then dichotomized into adequate (Score 1) or inadequate (Scores 0, 2, and 3). All data were registered daily. On all treatment days, each patient was analyzed and categorized according to the scores, so that 21 scores (days) were analyzed per patient.

## Color change

Subjective and objective methods were used to evaluate tooth color. Only the evaluators were unaware of the groups to which the participants had been assigned. Two examiners were trained on shade determination of eight subjects’ anterior teeth in a pilot study. This calibration was performed using Vita shade guide units (A1-D4) and a digital spectrophotometer (Vita Easyshade, Germany) made in the middle third of the upper and lower anterior teeth (incisors and canines), three times. For this, a custom tray was made with an orifice, with a 3.0mm radius window, to standardize the location of the color measuring. The color measurements were performed on the lateral and central incisors and on the canines, in both arches, with 12 teeth being evaluated per patient. The subjective evaluation was initially performed, followed by the objective evaluation. For the subjective evaluation, scores from 1 (B1) to 16 (C4) were assigned.^15,16^ A consensus was reached when no disagreements occurred.

Both measurements were performed at baseline (T0), seven (T1), fourteen (T2), and twenty-one days (T3) of treatment and the mean color change between evaluated teeth (incisors and canines) was calculated. A measurement was performed fourteen days after the completion of the treatment (T4). The color of each tooth was measured using the CIEDE2000 parameters: variables such as L*, a*, b*, h*, and c* coordinates that address not only L* (brightness), a* ( red-green), and b* (yellow-blue) found in the previous formula, but also the saturation (c*), hue angle (h*), matrix rotation term (RT), compensation for neutral colors; lightness compensation (SL), chroma compensation (SC), and hue compensation (SH), allowing a better correlation between the observed colors and bringing this score closer to what can be seen by the human eye. The shade comparison at different periods (T0, T1, T2, T3, and T4) was based on the ΔE00, which was calculated by the following formula:^17^


$$\begin{array}{cc} & \varepsilon \Delta E00(CIEDE2000)=\{[\Delta L/(KLSL)]2+[\Delta C/\\ & KCSC]2+[\Delta h/(KhSh)]2+\Delta R\}1/2\varepsilon \end{array}$$

## Tooth sensitivity

Tooth sensitivity was analyzed and measured from 0 to 10 in all periods, according to the visual analogue scale (VAS). Volunteers were asked about the intensity of tooth sensitivity caused by the treatment. A value of 0 was set when patients did not have any sensitivity and 10 when severe sensitivity occurred. In case of severe sensitivity, the patient would be excluded from the research and the dental bleaching treatment would be canceled.

## Statistical analysis

Statistical analysis was performed using a statistical software package (IBM SPSS Statistics 22.0; IBM Analytics, Armonk, NY, USA). The Kolmogorov-Smirnov test was used to evaluate the normality of the results. The chi-square test was used between the groups to compare the volunteers’ compliance behavior (usage time). The results for tooth color change and sensitivity were submitted to a two-way analysis of variance (ANOVA), followed by a Tukey post-hoc test. Statistical analysis was performed at a 5% significance level.

## Results

The 66 volunteers, aged 18 to 26 years (20±1.9 mean), were analyzed and a total of 462 readings (days of use) were performed for each study group, during the 21 days of treatment. No loss occurred during the follow-up periods.

### Daily compliance behavior degree

**Figure 3:**
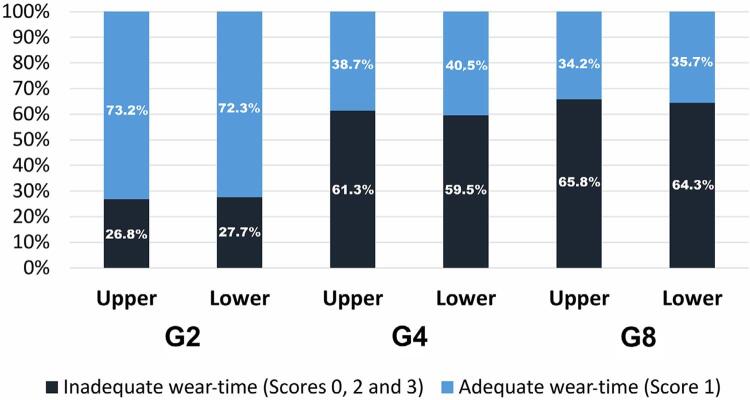
Dichotomized data regarding the usage time of the dental bleaching product for upper and lower arches for each group (n=462).

Data from daily compliance behavior is shown in Table 1 and Figure 3. A statistically significant difference was observed in the daily compliance behavior degree. G2 showed a greater cooperation degree than G8 (p<0.001). The lowest score 0 percentage was observed for G8. The highest score 1 percentage was observed for G2. Dichotomized data (adequate or inadequate) showed that the cooperation was inversely proportional to the recommended usage time (p<0.001).

**Table 1:** Distribution of patients’ 0-3 scores (usage time) according to each group

	Groups	Score 0	Score 1	Score 2	Score 3
Upper Arch	G2	59 (12.8%)	338 (73.2%)	26 (5.6%)	39 (8.4%)
	G4	60 (13.0%)	179 (38.7%)	50 (10.8%)	173 (37.4%)
	G8	24 (5.2%)	158 (34.2%)	132 (28.6%)	148 (32.0%)
Lower Arch	G2	59 (12.8%)	334 (72.3%)	28 (6.1%)	41 (8.9%)
	G4	60 (13.0%)	187 (40.5%)	46 (10.0%)	169 (36.6%)
	G8	25 (5.4%)	165 (35.7%)	131 (28.4%)	141 (30.5%)

### Color change

Table 2 shows the color changes obtained by the subjective evaluation of both arches. Significantly higher upper arch color change from T1 to T4 was observed in G8 than in G2; however, these differences were observed since T2 in the lower arch (p ≤0.05). ΔE00 values obtained by the objective analysis are shown in Table 3, comparing the periods to T0 (baseline). There were no statistical differences between the groups.

**Table 2:** Means (SD) of scores obtained by the subjective evaluation on upper and lower arches

		T0	T1	T2	T3	T4
Upper arch	G2	6.41 (±1.76)^Aa^	3.73 (±1.07)^Bb^	2.95 (±0.84)^Bc^	2.27 (±0.7)^Bd^	2.27 (±0.7)^Bd^
	G4	7.18 (±2.51)^Aa^	3.55 (±1.1)^ABb^	2.55 (±0.91)^ABc^	2.14 (±0.88)^ABd^	2.09 (±0.86)^ABd^
	G8	6.05 (±1.86)^Aa^	2.95 (±0.84)^Ab^	2.23 (±0.75)^Ac^	1.68 (±0.71)^Ad^	1.68 (±0.71)^Ad^
Lower arch	G2	5.86 (±1.47)^Aa^	3.55 (±0.91)^Ab^	2.86 (±0.77)^Bc^	2.41 (±0.66)^Bd^	2.41 (±0.66)^Bd^
	G4	6.86 (±2.64)^Aa^	3.5 (±1.05)^Ab^	2.55 (±0.85)^ABc^	2.23 (±0.86)^ABd^	2.09 (±0.86)^ABd^
	G8	5.95 (±1.96)^Aa^	2.95 (±0.84)^Ab^	2.23 (±0.75)^Ac^	1.73 (±0.76)^Ad^	1.73 (±0.76)^Ad^

Different uppercase letters in column and lowercase in row indicate statistically significant differences (p<0.05). Statistical analysis did not compare the upper arch with the lower arch.

**Table 3:** Means (SD) of color change (ΔE00) obtained by the objective evaluation on upper and lower arches

		T1	T2	T3	T4
Upper arch	G2	3.17 (±0.79)^Aa^	4.64 (±1.35)^Ab^	5.34 (±1.14)^Ab^	5.43 (±1.30)^Ab^
	G4	3.39 (±1.14)^Aa^	5.01 (±1.34)^Ab^	5.67 (±1.59)^Abc^	5.80 (±1.65)^Ac^
	G8	3.97 (±1.12)^Aa^	5.33 (±0.94)^Ab^	6.11 (±1.07)^Ab^	6.29 (±1.18)^Ab^
Lower arch	G2	2.87 (±0.68)^Aa^	4.22 (±1.13)^Ab^	4.71 (±1.30)^Ab^	4.60 (±1.35)^Ab^
	G4	2.92 (±0.82)^Aa^	4.17 (±1.06)^Ab^	4.72 (±1.36)^Ac^	4.82 (±1.36)^Ac^
	G8	3.38 (±1.38)^Aa^	4.07 (±1.09)^Ab^	4.89 (±1.56)^Ac^	4.81 (±1.41)^Ac^

Different uppercase letters in column and lowercase in row indicate statistically significant differences (p<0.05). Statistical analysis did not compare the upper arch with the lower arch.

For all groups, arches, and analysis, a significant and gradual increase in color change was observed (p ≤0.05), stabilizing at T4.

### Tooth sensitivity

Table 4 shows tooth sensitivity data. Only at T1 did G2 present significantly lower tooth sensitivity than G8 (p=0.047). Additionally, G2 showed higher sensitivity in T2 and T3 than in T1 and T4 (p≤0.05); however, only at T4 did lower tooth sensitivity than other time evaluations occur for G4 and G8.

**Table 4:** Means (SD) of scores obtained by VAS scale during and after the bleaching treatment

	T1	T2	T3	T4
G2	0.05 (0.21)^Aa^	0.77 (1.41)^Ab^	0.41 (0.85)^Ab^	0.09 (0.42)^Aa^
G4	0.82 (1.46)^ABb^	1.05 (1.52)^Ab^	1.36 (2.17)^Ab^	0.00 (0.00)^Aa^
G8	0.91 (1.41)^Bb^	0.82 (1.76)^Ab^	0.68 (1.49)^Ab^	0.00 (0.00)^Aa^

Different uppercase letters in column and lowercase in row indicate statistically significant differences (p <0.05).

## Discussion

This blinded, parallel, randomized clinical trial with similar allocation between treatment groups aimed to evaluate different aspects of bleaching treatment, such as daily compliance behavior, dental color change and tooth sensitivity. The main objective of this study was to assess compliance behavior daily—secondary outcomes are additional dependent variables, hierarchically less important.^18^ However, the study design and sample size were also based on the secondary outcomes (color change and sensitivity analysis), because results of these evaluations are related to usage time and daily compliance behavior.^19^

Based on the results of the present study, the first null hypothesis was rejected, because less usage time promoted more compliance of patients during at-home bleaching. Usage-time compliance behavior variations could possibly compromise the effectiveness of bleaching treatments. The effectiveness of an at-home dental bleaching procedures depends on tray usage time, which is entirely under the responsibility of the patient. Therefore, the clinician must guide the patient on the importance of properly following the recommended usage time in order to obtain effectiveness out of the treatment.^7-9^ The literature has reported on usage time of bleaching agents during at-home procedures.^2-5^ However, the present study is innovative because the usage time was evaluated using a direct measurement with electronic microsensors.^7-9^ It is important to highlight that no microsensors were lost in this study and that none of the volunteers reported discomfort using the trays containing the sensors.

A wide variability in usage time within some of the volunteers was observed. Drastic differences were found, with values ranging from 0 hours (Score 0) up to 12 hours and 15 minutes (Score 3), when the actual recommended usage time was 2 hours. This usage time pattern was a characteristic of patients who tried to compensate for little or no use time in a few days, or for those who used the trays while sleeping, for longer than recommended. Schott and Ludwig^10^ (2014) also found this compliance behavior when evaluating the usage time of removable orthodontic devices, documented microelectronically. These authors detected heterogeneous usage behavior, suggesting individualized treatment planning as an effective alternative for removable orthodontic treatments.^10^However, it is important to consider the degradation time of at-home products when recommending individual bleaching treatments.^20^ Calculating and tracking daily usage time may be a useful model for professionals and patients to assess the prescribed individual usage time of products during treatment, optimizing check-up appointments.^10^ The present study also demonstrated the importance of supervising volunteers’ compliance behavior degree during at-home dental bleaching, because the absence of this monitoring can lead to an incorrect interpretation of results, since the use of trays depends on the volunteer’s cooperation.

Although an 8-hour usage time is associated with use during sleep, a 2-hour usage time is more effective since it can be easily made at any time of the day. This finding is consistent with the medical literature, which suggests that less frequent dosing regimens are associated with better adherence.^21^

It was possible to observe, through subjective visual evaluation, higher color change for G8 than G2 at most of the evaluation time. For G2, even with higher patient compliance (score 1), reduced color change was observed, certainly due to the shorter usage time (2 hours). After 2 hours, more than 50% of the active agent is available in bleaching trays with reservoirs, and 19% in the ones without reservoirs.^6^ On the other hand, Marques, et al.^22^ (2012) found that, after 2 hours of use, only 7% of carbamide peroxide remains in trays. Longer exposure times to the bleaching product led to higher color change at each week of treatment, demonstrating that dental bleaching is time-dependent. Carbamide peroxide is easily broken down when saliva is maintained in contact with it, releasing hydrogen peroxide and urea, especially in bleaching trays during at-home protocols with low-concentration gels (10-22%).^23^ However, a greater presence of saliva inside the mandibular tray during treatment could have decreased the concentration of the bleaching gel and consequently, its efficacy,^23,24^ leading to results only being observed after T2. *In vivo* observations have pointed out that the rate of carbamide peroxide degradation during the bleaching process is twice as exponential and accelerated during the first hour of the process, and that 10% of the active agent is still available after 10 hours for trays with buccal reservoirs.^6^ It is important to mention that the trays were made without the presence of reservoirs, because reservoir and non-reservoir sides had equivalent color-change effects in a clinical trial with a one-year follow-up evaluation that used the same 10% carbamide peroxide gel as that of the present study.^25^

Spectrophotometry is an objective evaluation method capable of expressing minimal color differences in numerical form.^17,26^ It enables standardized color measurement, unlike the subjective method, in which color perception may be influenced by the adaptation of the dental color to the background area and the light source present.^27^ Formulas for calculating color differences that better represent the human perception have been developed, such as CIEDE2000 and CIELAB, although the second only agrees with visual findings in 75%.^17^ Statistical differences between G2 and G8 were only observed at T1. In this study, it was possible to observe a gradual increase/improvement in the color change from one-time analysis to the next evaluations, even after 14 days of the bleaching treatment.

Cardoso, et al.^2^ (2010), evaluating the use of 10% carbamide peroxide gel with objective and subjective analysis, found similar color changes for the 15-minute, 30-minute, and one-hour groups, statistically different from the changes of the eight-hour group. Corroborating these findings, the same product, when applied overnight, was more effective than applied 1 hour a day or for 20 minutes using 10% carbamide peroxide, according to objective analysis.^4,28^ Another clinical trial^5^ evaluated different at-home protocols and found higher color change in those who used the gel for 8 hours than in those who did so for 2 hours, only for the b coordinate, provided by objective analysis. In this context, at-home bleaching depends on time, not on concentration; therefore, there is no need for the use of high-concentration products: the number of days of treatment is more important.^29^

In the objective analysis, the difference between the upper arch means of groups G2 and G4 at T4 (0.86) can be correlated with the acceptability and perceptibility values proposed by Paravina, Pérez and Ghinea^17^ (2019), corresponding to the values in which 50% of evaluators/observers can detect a change in color. This result corroborates the subjective analysis, which also showed a statistical difference between G2 and G4.

Tooth sensitivity may be related not only to bleaching gel concentration, but also to its time of contact with dental structures.^2^ One of the mechanisms that cause tooth sensitivity is related to the penetration of peroxide into the pulp: the longer the contact time of the gel with the dental structure, the greater its chances of causing tooth sensitivity. The greater degree of collaboration of the volunteers with the use of the bleaching product and the shorter time of contact of the bleaching gel with the dental structure possibly contributed to G2 having presented less sensitivity after one week (T1). Mailart, et al.^5^ (2021) observed higher tooth sensitivity with 10% carbamide peroxide usage during 8 hours than during 2 hours, at 2 days of evaluation; however, no differences were found after 7 and 14 days. Other studies also found that using 28% carbamide peroxide for 20 minutes or 10% carbamide peroxide for 15, 30, and 60 minutes promoted less tooth sensitivity than using 10% carbamide peroxide overnight.^2,28^ However, other clinical trials observed a rate of 95% of no tooth sensitivity incidence for both 1 hour and overnight usages of 10% carbamide peroxide.^4^

However, in T2 and T3 the sensitivity means showed the same level of intensity, with results becoming practically null in T4, observations that are in agreement with other studies.^30^ Dental dehydration resulting from the use of trays for prolonged periods may be associated with a higher occurrence of tooth sensitivity.^31^ Thus, it has been suggested that prolonging the days of bleaching treatment is more indicated than using the trays for more hours.^29^ If the authors had discussed the daily compliance behavior with volunteers during the 21 days of treatment, inadequate daily usage time would probably have been avoided, which is a limitation of the present study. It is worth mentioning that other studies should be carried out evaluating different concentrations, bleaching products and color change stability in shorter, monitored usage times.

## Conclusions

Shorter recommended usage time of acetate trays/dental bleaching products improved patients’ compliance with the at-home dental bleaching treatment. However, increased daily usage time of the bleaching gel promoted better subjective color change. For a longer usage time, tooth sensitivity was more significant in the first week.
